# Intracranial Metastatic Disease: Present Challenges, Future Opportunities

**DOI:** 10.3389/fonc.2022.855182

**Published:** 2022-03-07

**Authors:** Alyssa Y. Li, Karolina Gaebe, Katarzyna J. Jerzak, Parneet K. Cheema, Arjun Sahgal, Sunit Das

**Affiliations:** ^1^ Institute of Medical Science, Faculty of Medicine, University of Toronto, Toronto, ON, Canada; ^2^ Division of Oncology, Department of Medicine, Sunnybrook Health Sciences Centre, University of Toronto, Toronto, ON, Canada; ^3^ Division of Oncology, William Osler Health System, Brampton, ON, Canada; ^4^ Department of Radiation Oncology, Sunnybrook Health Sciences Centre, University of Toronto, Toronto, ON, Canada; ^5^ Division of Neurosurgery, St. Michael’s Hospital, University of Toronto, Toronto, ON, Canada

**Keywords:** intracranial metastatic disease (IMD), brain metastases, targeted therapy, immunotherapy, neurosurgery, minimally invasive surgery, radiation therapy, screening

## Abstract

Intracranial metastatic disease (IMD) is a prevalent complication of cancer that significantly limits patient survival and quality of life. Over the past half-century, our understanding of the epidemiology and pathogenesis of IMD has improved and enabled the development of surveillance and treatment algorithms based on prognostic factors and tumor biomolecular characteristics. In addition to advances in surgical resection and radiation therapy, the treatment of IMD has evolved to include monoclonal antibodies and small molecule antagonists of tumor-promoting proteins or endogenous immune checkpoint inhibitors. Moreover, improvements in the sensitivity and specificity of imaging as well as the development of new serological assays to detect brain metastases promise to revolutionize IMD diagnosis. In this review, we will explore current treatment principles in patients with IMD, including the emerging role of targeted and immunotherapy in select primary cancers, and discuss potential areas for further investigation.

## 1 Introduction

The development of intracranial metastatic disease (IMD) is a frequent and serious complication of cancer, affecting nearly 30% of cancer patients (1). The incidence of IMD is expected to increase as our population ages and as advancements in primary cancer therapy result in longer disease survival (1). IMD has been observed to disproportionately affect patients with certain primary cancers, including lung cancer (19.6%), melanoma (6.4%), renal cell carcinoma (4.2%), breast cancer (3.1%), and colorectal cancer (1.4%), reflecting possible organotropy in IMD or successes with local disease control (2). Indeed, among patients with brain metastases (BrM), lung cancer, breast cancer, and melanoma account for nearly 60%, 11%, and 6% of all primary cancers in some studies (2).

The impact of IMD on morbidity and mortality of patients with cancer is significant. The median time to diagnosis of IMD has been reported to be 5.2 months following primary cancer diagnosis, suggesting that a large proportion of patients may already have BrM at the time their primary cancer is diagnosed (2). Given the high prevalence of IMD and the short median survival of 3.7 months following IMD diagnosis, there is interest in developing screening strategies and tools to identify patients with IMD (2). Magnetic resonance imaging (MRI) remains the most commonly used modality for the diagnosis of BrM and leptomeningeal disease (3). Historically, MRI evaluation has been prompted by the development of neurological symptoms, including headache, seizure, and altered mental status, in patients with cancer. Several algorithms have since been created to identify asymptomatic, high-risk patients who would benefit from MRI screening at the time of their primary cancer diagnosis (3). Imaging and serum biomarkers of IMD to allow for earlier and more cost-effective diagnosis of IMD are under investigation, however, their role in IMD surveillance remains to be fully elucidated (4, 5).

At present, the most common local treatments for IMD are surgical resection, whole brain radiation therapy (WBRT), and stereotactic radiosurgery (SRS). The Graded Prognostic Assessment (GPA) is often used to estimate survival for patients with BrM and is based on prognostic factors including Karnofsky performance score (KPS), age, presence of extracranial metastases, number of BrM, and tumor subtype, depending on the primary tumor histology (6). In some treatment algorithms, patients with greater IMD burden or poorer performance status are directed toward WBRT, while younger patients with fewer intracranial metastases may be directed toward treatment with surgical resection or SRS (7). Advances in surgical technique and delivery of radiation have resulted in improved survival estimates for patients with IMD from less than six months over fifteen years ago to 8-16 months today (6, 8–11).

Recent data have supported a role for systemic targeted therapies and immunotherapies in the treatment of select patients with IMD, including but not limited to those with non-small cell lung cancer (NSCLC), breast cancer, and malignant melanoma (12, 13). There remains, however, a clear clinical need for further investigation to develop more effective screening and treatment for IMD. In this review, we outline and describe recent and current efforts to improve outcomes in patients with IMD of the brain through novel therapeutics, improved surveillance, and prevention.

## 2 Emerging Treatments for IMD

Traditionally, prognosis for patients with IMD has been extremely poor. However, modern treatments have significantly improved survival and quality of life (QoL) in these patients. The main goals of IMD therapy include lengthening survival, diminishing or controlling IMD burden, minimizing adverse events associated with IMD development and treatment, as well as improving cognition and QoL.

### 2.1 Surgical Resection and Minimally Invasive Surgery

Surgery has been a long-standing modality in the treatment of BrM. In contrast to other forms of IMD management, surgical resection can offer immediate relief of mass effect and neurological symptoms caused by compression and edema, while providing tissue for diagnostic purposes (14, 15). Risks associated with neurosurgery include iatrogenic neurological injury, hemorrhage, infection, and seizure (16, 17). Recent literature has also confirmed a long-standing concern that surgery holds a risk of leptomeningeal disease recurrence, likely related to unintentional local cell seeding during the process of surgical resection (18). However, with modern techniques, surgical resection is typically safe and recovery from surgery often short, making surgery an appealing option, particularly for patients with a large brain lesion, limited number of BrM, and well controlled systemic disease (19). In the 1990s, two landmark randomized controlled trials (RCTs) demonstrated that surgical resection compared with needle biopsy or no surgery prior to WBRT decreased local recurrence, improved median survival, and resulted in more rapid and sustained functional independence (20, 21). In contrast, a similar study by Mintz et al., which included many patients with progressive extracranial disease and lower performance status, found no survival benefit with the addition of surgical resection to WBRT (22). A recent systematic review and meta-analysis found that the literature supports surgical resection followed by postoperative WBRT in patients with good performance status (KPS ≥ 70), controlled systemic disease, and a single brain lesion (23).

While surgical resection of a single BrM in patients with well controlled systemic disease is well-established in clinical practice, the role of surgery in the treatment of patients with limited BrM (2-4 lesions) or deep BrM remains a topic of debate (22, 24). In current clinical practice, these patients are more often treated with SRS. A few retrospective studies have reported equivalence of outcomes between groups treated with surgery and SRS for limited BrM (25–27). SRS is less effective for larger lesions and is associated with delayed treatment effect, differences that have become more significant as improvements in systemic therapies have led to prolonged overall survival (OS) and highlighted the importance of achieving adequate IMD control to maintain QoL (24, 28). These factors, as well as the significant benefits observed with surgical resection in the management of single brain lesions, have prompted investigation into the role of surgery in patients with limited BrM or deep, large lesions (29–31).

Advances in surgical technique and intraoperative technologies may expand indications for surgical resection. Improvements in microsurgery, for example, through improved microscopic and endoscopic visualization, the use of “keyhole” techniques, and evolution away from the use of fixed retractors during surgery, have greatly reduced surgical morbidity and mortality, with recent studies reporting a complication rate between 2-9% (32–36). Further, the development of minimally invasive parafascicular surgery (MIPS), based on the use of a minimal-access tubular retractor advanced into the brain using stereotactic guidance, has allowed surgeons access to deep-seated lesions, while minimizing the iatrogenic injury that has been historically the cost of reaching these areas (37). Even in the setting of high-risk brain lesions, minimal complications were reported by Gassie et al. with the use of MIPS in 15 patients with deep-seated brain tumors: only one patient had decreased postoperative KPS and no patients developed local complications, such as stroke, infection, hemorrhage, or seizure (38). These results imply that surgical management of deep-seated, high-risk brain lesions may be feasible, however, clinical efficacy in comparison with other treatment approaches remains to be studied.

### 2.2 Radiation Therapy

WBRT has been a mainstay for the treatment of IMD since the middle of the 20^th^ century (39–41). Gains in patient survival with improvements in systemic therapies, however, have brought mounting concerns regarding the late toxicities of WBRT, particularly on neurocognitive function (42). Several modifications to the delivery of WBRT have since been investigated, for example, techniques that spare exposure to the hippocampus, or concurrent delivery of a neuroprotective agent (43–46). In current practice, WBRT remains the preferred treatment option for patients with multiple BrM, widely disseminated metastatic disease, or short life expectancy, and is used as prophylactic therapy for patients with small cell lung cancer (SCLC) in the absence of IMD (47).

#### 2.2.1 The Evolution of SRS in the Treatment of IMD

A major improvement in the treatment of BrM has been SRS, which allows for focal delivery of high doses of radiation. For patients with limited (1–4) BrM, SRS has been shown to be an effective treatment to achieve local control of lesions within the cerebrum, cerebellum, and brainstem (48, 49). A secondary analysis of an RCT designed to investigate the addition of WBRT to SRS or surgical resection showed similar rates of local control following surgical resection and WBRT, compared with WBRT followed by cavity radiation with SRS (50). At present, we do not have RCT data that directly compare surgical resection and SRS in the setting of a single or limited BrM. Multiple studies, however, have investigated 1) the utility of adding SRS to WBRT; and 2) treatment with SRS as an alternative to WBRT.

Two seminal RCTs by Andrews et al. and Kodziolka et al. investigated the benefit of SRS boost following WBRT for patients with limited or multiple BrM. Both trials found that SRS improved OS (6.5 vs. 4.9 months, p=0.39; and 11.0 vs. 7.5 months, p=0.03) as well as local control (51, 52). Given the neurotoxicity associated with WBRT, Aoyama et al. then asked if SRS was sufficient as a treatment for patients with limited BrM. One hundred thirty-two patients were randomized to SRS alone (n=65) or WBRT and SRS (n=67). Although higher rates of local tumor control were observed at 1 year in the group treated with WBRT and SRS compared with standalone SRS treatment (88.7% vs 72.5%, p=0.002), the addition of WBRT did not confer a statistically significant benefit on survival (median 7.5 vs. 8.0 months, p=0.42) (53). A subsequent RCT by Chang et al. to evaluate neurocognitive decline in patients with limited BrM treated with SRS or WBRT plus SRS was halted prematurely when an interim analysis showed that patients randomized to receive WBRT plus SRS were at a significantly increased risk for neurocognitive decline at four months (54). Notably, their findings again showed a dissociation between achievement of local control and OS: both 1-year freedom from recurrence and risk of death were higher with combined WBRT and SRS (hazard ratio (HR) 2.47, p=0.0036; 27% vs. 73%, p=0.0003). The finding in this study that treatment with SRS alone conferred a greater survival benefit than bimodality treatment has received criticism as an artifact of higher burden of extracranial and intracranial disease in the WBRT plus SRS arm (55). Overall, these studies highlight the fact that many of these patients succumb to progression of their systemic disease.

A study by the European Organization for Research and Treatment of Cancer (EORTC) similarly found that WBRT improved local control at 2 years compared with observation only after SRS (31% vs. 19%, p=0.040). In line with the findings by Aoyama and colleagues, however, no OS difference was observed between the SRS alone and WBRT plus SRS arms (SRS only: 10.7 months, WBRT plus SRS: 10.9 months, p=0.89). Further, improved tumor control with the addition of WBRT did not lengthen the median duration of functional independence (SRS only: 10.0 months, WBRT plus SRS: 9.5 months, p=0.71) (56). In fact, QoL was better in the SRS-only arm (57). In a separative analysis of this trial, Kim et al. found that, compared with upfront WBRT, SRS alone was more cost-effective in patients with one to three IMD lesions (58). Meta-analyses by Tsao et al. and Sahgal et al. concluded that, while WBRT improved local and distant brain control compared with SRS alone (HR 2.61, p<0.0001, and HR 2.15, p<0.00001, respectively), treatment with SRS alone is associated with longer survival in patients age ≤ 50 years (13.6 vs. 8.2 months), and that there is no difference in survival with the addition of WBRT to SRS in patients age > 50 years, compared to SRS alone (10.1 vs. 8.6 months, respectively) (59, 60). A subsequent trial in which patients with limited melanoma BrM treated with SRS were randomized to adjuvant WBRT showed no clinical benefit with the addition of WBRT to local therapy on distant intracranial control, survival, or preservation of performance status (61). Consequently, in a statement released by the American Society for Radiation Oncology as part of its Choosing Wisely campaign, addition of WBRT to SRS is no longer recommended for patients with limited number of BrM (62). Conversely, WBRT remains standard of care in patients with multiple (≥ 4 BrM). More recent data have supported a possible role for SRS in the treatment of patients with multiple (≤ 10) BrM. A prospective observational study of nearly 1200 Japanese patients found similar rates of OS in patients treated with SRS for two to four lesions compared with SRS for five to ten lesions (HR 0.79, p=0.78) (63).

#### 2.2.2 Late Adverse Effects Following SRS

Although SRS is generally well-tolerated, patients can experience adverse effects, most notably radiation necrosis, which negatively affects QoL and morbidity. Radiation necrosis develops in 5-25% of patients following SRS treatment and constitutes a late adverse treatment effect that can occur months to years after SRS has been completed (64–70). Patients affected by radiation necrosis can present with a variety of symptoms ranging from asymptomatic to symptoms of increased intracranial pressure, including headaches, seizures, or cognitive/neurological decline (71). Diagnosis of radiation necrosis is challenging, as radiation necrosis and recurrent tumor lesions present with similar features on conventional imaging. Ultimately, biopsies of suspicious lesions are required to establish a definite diagnosis. While glial cell and vascular injury have been postulated as potential underlying etiologies for radiation necrosis, damage following SRS is directly related to radiation dose per fraction administered (72–74). Additional risk factors include prior radiation (in particular SRS) to the same site, larger lesion size, or receipt of targeted or immunotherapy (64, 75–77). In one study, SRS to the same lesion was associated with an increased risk of radiation necrosis at 1 year when compared with prior WBRT, concurrent WBRT, or no prior radiation (20%, 4%, 8%, and 3%, respectively) (77). Blonigen et al. also demonstrated that the risk of radiation necrosis was higher when the volume of brain parenchyma receiving more than 10 Gy or 12 Gy exceeded 10.5 cm^3^ or 7.9 cm^3^, respectively (64). In another study of 480 patients secondary to NSCLC, melanoma, or renal cell carcinoma, the risk of radiation necrosis following SRS treatment was 2.5 times higher in patients who received prior immunotherapy, further illustrating the multi-faceted nature of radiation necrosis (78). Management of radiation necrosis can be conservative in the absence of symptoms, or can involve corticosteroids, bevacizumab, surgical resection, or laser interstitial therapy for symptom control (79–82). Radiation necrosis is a challenging complication of SRS treatment and should be considered as a potential complication following SRS.

#### 2.2.3 Approaches to Limit Cognitive Impacts of WBRT

Given the neurotoxicity associated with WBRT, several strategies to limit the cognitive impacts of WBRT have been investigated. Our advancing understanding of the mechanisms underlying radiation-induced injury has led to the development of pharmacological agents that modulate the brain’s sensitivity to radiation-induced effects. One such agent is memantine, a drug that is used in the treatment of Alzheimer’s dementia and that helps prevent vascular injury. The efficacy of memantine in preventing radiation-induced cognitive dysfunction was investigated in a RCT (RTOG 0614) of 508 patients treated with WBRT and either memantine (20 mg/day) or placebo, both initiated within three days of WBRT for 24 weeks. There were no differences in OS and progression-free survival (PFS) between the two arms, and memantine did not cause additional toxicity. Although no statistically significant differences in terms of the Hopkins Verbal Learning Test–Revised Delayed Recall (HVLT-R DL) were observed at 24 weeks (memantine median decline: 0, placebo median decline: -2, p=0.059), participants treated with memantine showed delayed time to cognitive decline (HR 0.78, 95% CI 0.62-0.99, p=0.01) and superior executive function at 8 (p=0.008) and 16 weeks (p=0.0041), as well as superior processing speed (p=0.0137) and delayed recognition (p=0.0149) at 24 weeks (43). Following these results, the National Cancer Comprehensive Network (NCCN) incorporated the use of memantine along with WBRT into their consensus guidelines. Despite this, routine use of memantine for patients receiving WBRT has not yet been established; future health policy strategies should develop ways in which memantine can be more quickly adopted into routine therapy (83). Other agents, such as the donepezil, renin-angiotensin system blockers, or *Ginkgo biloba*, have also been investigated (84–87).

The decline in memory function observed following WBRT may be a consequence of radiation-induced injury to the hippocampus, a region which is involved in neurocognitive functions, including memory, learning, and spatial information processing (88). Given the low frequency of BrM in the hippocampus, this region could represent a dose-limiting structure (89). Early data to support this hypothesis stems from a single-arm, multi-institutional phase II study (RTOG 0933) that showed superior cognitive preservation (as measured by the HVLT-R DR) with the use of hippocampal avoidance WBRT (HA-WBRT) compared with historical data from WBRT-treated controls (mean relative decline from baseline to 4 months 7% vs. 30%, p<0.001) (45).

Given the success observed with memantine, a more recent phase III trial investigating the addition of memantine to HA-WBRT showed significantly lower risk of cognitive failure after HA-WBRT plus memantine compared with HA-WBRT alone (HR 0.74, 95% CI 0.58-0.95) (90). Treatment efficacy in terms of intracranial PFS (iPFS) and OS did not differ between the two arms, suggesting that HA-WBRT plus memantine should be considered as standard of care for patients scheduled to receive WBRT with no BrM in the hippocampal region. As patients experience longer survival, neurological sequalae from radiation treatment will become increasingly important. Anatomical avoidance and pharmacotherapy are promising ways for clinicians to preserve cognitive function in patients receiving WBRT.

#### 2.2.4 Radiation Therapy in the Setting of SCLC

WBRT does remain the standard of care for patients with SCLC in the form of prophylactic cranial irradiation (PCI). As many as 40-60% of patients with SCLC will develop IMD during the course of their disease, with rates of IMD 1.3-2 times higher than in patients with NSCLC (91). Notably, this standard is being challenged by mounting evidence from retrospective studies and meta-analyses on the lack of survival benefit in the extensive stage setting and additional neurotoxicity of PCI in patients with SCLC (92–94). As patients with SCLC have been historically excluded from trials for SRS, data comparing SRS and WRBT alone, or SRS and WBRT + boost, in this patient population is uniformly retrospective. A propensity score-matched observational study by Rusthoven et al. found that, as for other malignancies, the addition of WBRT to SRS improved intracranial control (time to central nervous system (CNS) progression HR 0.28, p<0.001), but not survival (median OS 5.2 months for WBRT vs. 6.5 months for SRS, p=0.79), in patients with SCLC (95). Similarly, a phase III trial found equivalent OS and intracranial control with WBRT with hippocampal sparing (HA-WBRT) compared to conventional WBRT, with prolonged preservation of neurocognitive function in the HA-WBRT arm (96). These findings suggest a trend away from conventional WBRT as PCI for patients with SCLC toward treatments aligned to achieve IMD control while maintaining QoL and cognition.

### 2.3 Pharmacological Therapies for IMD

Although surgery and radiation therapy remain the cornerstone of treatment for patients with IMD, recent data support a role for targeted systemic therapies and immunotherapy with immune check point inhibitors in certain patient subgroups.

#### 2.3.1 Targeted Therapies

Advances in genetic and genomic analyses have enabled the discovery of genetic alterations that promote tumor growth and proliferation. In a subset of patients with breast cancer, for example, amplification of the human epidermal growth factor receptor 2 (HER2/neu) has been shown to drive cancer propagation (97–99). The development of small molecule or antibody-based agents to target these molecular drivers of cancer and their associated signalling pathways has revolutionized the treatment of patients with HER2-positive breast cancer, epidermal growth factor receptor (EGFR)-mutant or anaplastic lymphoma kinase (ALK)-rearranged NSCLC, and BRAF-mutant melanoma (**Table 1**).

**Table 1:** Summary of studies investigating targeted therapies for IMD secondary to breast cancer, NSCLC, and melanoma.

	Drug	Trial/Study	Study design	Total participants (n)	Study arms	Median OS (months)	Findings
Breast cancer	Trastuzumab	Slamon et al. (100)	RCT	469	Standard chemotherapy ± trastuzumab in all breast cancer patients	25.1 vs. 20.3 (p=0.046)	Relative risk reduction of death at 30-month follow-up: 20%
		Park et al. (101)	Retrospective cohort study	251	Palliative chemotherapy ± trastuzumab in all breast cancer patients	31.7 vs. 16.7 (p=0.001)	Incidence of BrM: 37.8 vs. 25% (p=0.028) TTD from BrM: 14.9 vs. 4.0 months (p=0.0005)
		Park et al. (102)	Retrospective cohort study	78	Trastuzumab after BrM diagnosis vs. trastuzumab before BrM diagnosis only vs. no trastuzumab	13.6 vs. 5.5 vs. 4.0 (p<0.001)	Median TTP of BrM: 7.8 vs. 3.9 vs. 2.9 months (p=0.006) HR for death in patients with BrM: 0.5 (p=0.017)
		Okita et al. (103)	Retrospective cohort study	62	Trastuzumab vs. no trastuzumab	38.4 vs 8.4 (p=0.0005)	Median second brain metastatic-free survival time: 7.0 vs. 5.6 months (p=0.057)
		Dawood et al. (104)	Retrospective cohort study	598	Trastuzumab vs. no trastuzumab vs. HER2-negative	11.6 vs. 6.1 vs. 6.3 (p<0.0001)	–
	Lapatinib	Lin et al. (105)	Single-arm clinical trial	39	Lapatinib	–	CNS ORR: 2.6%
		Lin et al. (106)	Single-arm clinical trial	242	Lapatinib, lapatinib and capecitabine (n = 50)	–	CNS ORR: 6% (lapatinib alone), 20% (with capecitabine) ≥20% BrM volume reduction: 21% (lapatinib alone), 40% (with capecitabine)
		Metro et al. (107)	Retrospective cohort study	30	Lapatinib and capecitabine	27.9 vs. 16.7 (p=0.01)	CNS ORR: 31.8% Disease stabilization: 27.3%
		Bachelot et al. (108)	Single-arm clinical trial	45	Lapatinib and capecitabine	17.0	Median TTP: 5.5 months CNS ORR: 65.9% Disease stabilization: 36%
	Neratinib	Freedman et al. (109)	Single-arm clinical trial	49	Neratinib and capecitabine in lapatinib-naïve and lapatinib-treated patients	13.3 and 15.1	CNS ORR: 49% and 33% Median PFS: 5.5 and 3.1 months
		Hurvitz et al. (110)	RCT	101	Neratinib and capecitabine vs. lapatinib and capecitabine	13.9 vs. 12.4 (p=0.635)	Median PFS: 5.6 vs. 4.3 months (p=0.074)
	Tucatinib	Lin et al. (111)	RCT	291	Trastuzumab and capecitabine with or without tucatinib	18.1 vs. 12.0 (p=0.005)	Median PFS: 9.9 vs. 4.2 months, HR 0.32, P<0.0001 iORR: 47.3% vs. 20%, p=0.03
	Trastuzumab emtansine (T-DM1)	Bartsch et al. (112)	Retrospective cohort study	10	T-DM1	–	iORR: 30%
		Jacot et al. (113)	Single-arm clinical trial	2002	T-DM1	–	–
		Krop et al. (114)	RCT	991	T-DM1 vs. capecitabine and laptinib	26.8 vs. 12.9 (HR 0.38, p=0.008)	Median PFS: 5.9 vs. 5.7 months (HR 1.00, p=1.0)
		Montemurro et al. (115)	Single-arm clinical trial	2002	T-DM1	18.9	Median PFS: 5.5 months ORR: 21.4%
	Trastuzumab deruxtecan (T-DXd)	Barsch et al. (116)	Single-arm clinical trial	10	T-DXd	–	CNS ORR: 83.3%
		Jerusalem et al. (117)	Single-arm clinical trial	24	T-DXd	–	Median PFS: 18.1 months ORR: 58.3% CNS ORR: 50%
	Abemaciclib	Tolaney et al. (118)	Non-randomized clinical trial	104	Abemaciclib ± hormone therapy	12.5	CNS ORR: 5.2%
					Abemaciclib and trastuzumab	10.1	CNS ORR: 0% Median intracranial PFS: 2.7 months
	Palbociclib	Brastianos et al. (119)	Single-arm clinical trial	15	Palbociclib	6.4	Intracranial disease benefit rate: 53.3%
	Iniparib	Anders et al. (120)	Single-arm clinical trial	37	Iniparib and irinotecan	7.83	CNS ORR: 12%
	Talazoparib	Litton et al. (121)	RCT	431	Talazoparib vs. chemotherapy	– (HR 0.671, 95% CI 0.366-1.229)	–
NSCLC	Crizotinib	Solomon et al. (122)	RCT	343	Crizotinib vs. pemetrexed + platinum-based chemotherapy	–	Median PFS: 9.0 vs. 4.0 months, HR 0.40, P<0.001 iORR: 77% vs. 28%, p<0.001
	Ceritinib	Crinò et al. (123)	Single-arm clinical trial	140	Ceritinib	–	Median PFS: 5.4 months Intracranial ORR: 33%
	Alectinib	Gadgeel et al. (124)	Single-arm clinical trial	47	Alectinib	–	iORR: 52%
		Peters et al. (125)	RCT	303	Alectinib vs. crizotinib	–	PFS rate: 12% vs. 45%, HR 0.51, p<0.001
	Brigatinib	Camidge et al. (126)	RCT	275	Brigatinib vs. crizotinib	–	12-month PFS rate: 67% vs. 21%, HR 0.27 Intracranial median TTP: HR 0.30 iORR: 78% vs. 29%, OR 10.42
	Lorlatinib	Shaw et al. (127)	RCT	296	Lorlatinib vs. crizotinib	–	12-month PFS rate: 96% vs. 60%, HR 0.07 iORR: 82% vs. 23%, OR 16.83
		Shaw et al. (128)	Single-arm clinical trial	364	Lorlatinib	–	CNS ORR (TKI-naive): 64% CNS ORR (previous crizotinib): 50%
	Ensartinib	Horn et al. (129)	RCT	290	Ensartinib vs. crizotinib	–	Median PFS (baseline BrM): 11.8 vs. 7.5 months (HR 0.55, p=0.05) Median PFS (no baseline BrM): NR vs. 16.6 months (HR 0.46, p=0.003)
	Lazertinib	Ahn et al. (130)	Single-arm clinical trial	127	Lazertinib	–	CNS ORR: 44%
		Cho et al. (131)	Single-arm clinical trial	78	Lazertinib	–	CNS ORR: 85.7%
	Furmonertinib	Shi et al. (132)	Single-arm clinical trial	130	Furmonertinib	–	Median PFS: 9.9 months CNS ORR: 58.8%
		Shi et al. (133)	Single-arm clinical trial	220	Furmonertinib	–	CNS ORR (measurable BrM): 66% CNS ORR (measurable/non-measurable BrM): 34% Median PFS (measurable/non-measurable BrM): 11.6 months
	Amivantamab	Park et al. (134)	Single-arm clinical trial	81	Amivantamab	–	ORR: 39%
	Gefitinib	Ceresoli et al. (135)	Single-arm clinical trial	41	Gefitinib	–	Median PFS: 3.0 months iORR: 10% DCR: 27%
		Hotta et al. (136)	Retrospective cohort study	57	Gefitinib	–	iORR: 42.9%
		Lee et al. (137)	Single-arm clinical trial	37	Gefitinib	–	iORR: 70%
		Chiu et al. (138)	Single-arm clinical trial	76	Gefitinib	–	iORR: 33.3% DCR: 63.2%
		Kim et al. (139)	Double-arm clinical trial	23	Gefitinib or erlotinib	18.8	Median PFS: 7.1 months iORR: 73.9% Overall ORR: 69.6% Overall DCR: 82.6%
		Park et al. (140)	Double-arm clinical trial	28	Gefitinib or erlotinib	15.9	Median PFS: 6.6 months Overall ORR: 83% Overall DCR: 93%
	Osimertinib	Mok et al. (141)	RCT	419	Osimertinib vs. pemetrexed with platinum-based chemotherapy	–	Median PFS: 8.5 vs. 4.2 months, HR 0.32
		Soria et al. (142)	RCT	456	Osimertinib vs. erlotinib or gefitinib	–	Median PFS: 15.2 vs. 9.6 months, HR 0.47, p<0.001
	Sotorasib	Skoulidis et al. (143)	Single-arm clinical trial	126	Sotorasib in all KRAS^G12C^-positive patients	12.5	Median PFS: 6.8 months Overall ORR: 37.1%
	Selpercatinib	Drilon et al. (144)	Single-arm clinical trial	105	Selpercatinib	–	CNS ORR: 91%
	Pralsetinib	Gainor et al. (145)	Single-arm clinical trial	233	Pralsetinib	–	CNS ORR: 56%
	Repotrectinib	Drilon et al. (146)	Single-arm clinical trial	–	Repotrectinib	–	–
	Tepotinib	Paik et al.	Single-arm clinical trial	152	Tepotinib	–	Median PFS: 10.0 months ORR: 55%
	Capmatinib	Wolf et al.	Single-arm clinical trial	364	Capmatinib	–	CNS ORR: 53.8%
	Laprotrectinib	Hong et al.	Single-arm clinical trial	159	Laprotrectinib	–	CNS ORR: 66.7%
	Entrectinib	John et al.	Single-arm clinical trial	16	Entrectinib	–	CNS ORR (measurable BrM): 62.5% CNS ORR (measurable/non-measurable BrM): 50%
Melanoma	Dabrafenib	Long et al. (147)	Single-arm clinical trial	172	Dabrafenib in BRAF^V600E^-positive melanoma patients with treatment-naïve IMD or progressive IMD	7.64 and 7.25	Median PFS: 3.72 and 3.83 months iORR: 39.2% and 30.8%
		Davies et al. (148)	Single-arm clinical trial	125	Dabrafenib and trametinib in BRAF^V600E^-positive melanoma patients with treatment-naïve IMD or progressive IMD	10.8 and 24.3	Median PFS: 5.6 and 7.2 months iORR: 58% and 56%
	Vemurafenib	McArthur et al. (149)	Single-arm clinical trial	146	Vemurafenib in BRAF^V600^-positive melanoma patients with treatment-naïve IMD or progressive IMD	8.9 and 9.6	Median PFS: 3.7 and 4.0 months iORR: 18% and 18%

Median overall survival marked with a dash if the data was 1) not reported or 2) reported for the entire population, including patients without IMD.

BrM, brain metastases; CI, confidence interval; CNS, central nervous system; DCR, disease control rate; HER2, human epidermal growth factor 2; HR, hazard ratio; IMD, intracranial metastatic disease; iORR, intracranial ORR; NR, not reached; NSCLC, non-small cell lung cancer; OR, odds ratio; ORR, objective response rate; OS, overall survival; PFS, progression-free survival; RCT, randomized control trial; T-DM1, trastuzumab emtansine; T-DXd, trastuzumab deruxtecan; TKI, tyrosine kinase inhibitor; TTD, time to death; TTP, time to progression.

##### 2.3.1.1 Targeted Therapies in Breast Cancer

As IMD is a frequent complication in patients with breast cancer, a large body of literature is available that describes current efforts to identify and evaluate targeted therapy in HER2-positive breast cancer, hormone receptor (HR)-positive breast cancer, and triple negative breast cancer (TNBC).

###### 2.3.1.1.1 HER2-Positive Breast Cancer

The addition of trastuzumab, a monoclonal antibody against the HER2 receptor, has been shown to prolong OS and PFS in patients with HER2-positive breast cancer (100). While trastuzumab therapy has been demonstrated to improve systemic disease control, population-based studies identified an increased incidence of IMD in patients treated with trastuzumab, likely resulting from the prolongation in survival and limited penetration of drug across the blood-brain barrier (BBB), rendering the brain a “sanctuary site” for cancer cells (150–152). Notably, HER2-positive breast cancer patients treated with palliative chemotherapy and trastuzumab were more likely to develop BrM than patients who received palliative chemotherapy alone (37.8% vs. 25.0%, p=0.028); conversely, median time to death (TTD) measured from the development of IMD was significantly longer for patients treated with palliative chemotherapy and trastuzumab, compared to those treated with palliative chemotherapy alone (14.9 vs. 4.0 months, p=0.0005), suggesting that trastuzumab might exert some biological effect, even if partial, on IMD (101). Supporting this hypothesis, studies have similarly found that trastuzumab prolongs median OS in HER2-positive breast cancer patients with IMD (102–104).

Lapatinib, an inhibitor of HER2 and EGFR, was subsequently developed to treat HER2-positive breast cancer that had progressed on all previous lines of therapy. From the perspective of IMD, lapatinib was theorized to have better BBB penetrance than trastuzumab, given its smaller molecular weight. Initial phase II trial results in HER2-positive breast cancer patients with progressive IMD despite WBRT or SRS demonstrated modest antitumor activity with either lapatinib alone (CNS objective response rate (ORR): 2.6-6%) or in combination with capecitabine (CNS ORR: 20%) (105, 106). Subsequent work has suggested that the combination of lapatinib and capecitabine increases median OS from the time of IMD development in HER2-positive breast cancer patients previously treated with anthracycline, trastuzumab, and a taxane, compared with patients receiving anthracycline, trastuzumab, and a taxane only (27.9 vs. 16.7 months, p=0.01) (107). The combination of lapatinib and capecitabine also demonstrated high antitumor activity (CNS ORR: 65.9%) in HER2-positive breast cancer patients with previously untreated IMD in the LANDSCAPE trial, though the impact of treatment on OS was not explicitly determined and concerns regarding treatment toxicity and delays in accessing radiotherapy were raised (108).

Following the development of lapatinib, additional small molecule candidates have been engineered. Neratinib, an irreversible inhibitor of HER1, HER2, and HER4, has been demonstrated to have good antitumor activity in combination with capecitabine in lapatinib-naive or lapatinib-treated patients with IMD secondary to HER2-positive breast cancer (CNS ORR: 49% and 33%, respectively). Among these two patient cohorts, median PFS was 5.5 and 3.1 months, respectively, and median OS was 13.3 and 15.1 months, respectively, though no direct comparisons between groups were reported (109). When compared head-to-head with lapatinib and capecitabine (L+C) in a recent RCT, neratinib and capecitabine (N+C) showed a substantial though not statistically significant effect on OS (13.9 vs. 2.4 months, HR 0.90, p=0.635) and PFS (5.6 vs. 4.3 months, HR 0.66, p=0.074) in HER2-positive breast cancer patients with IMD who have previously failed at least two anti-HER2 therapies (110). The lack of statistical significance may have been due to small sample sizes. Of 621 patients enrolled, 101 (16.3%) had known CNS metastases at baseline (N+C: *n* = 51; L+C: *n* = 50); 81 had received prior CNS-directed radiotherapy or surgery. In the CNS subgroup, mean PFS through 24 months was 7.8 months with N+C versus 5.5 months with L+C (HR 0.66, 95% CI 0.41–1.05), and mean OS through 48 months was 16.4 vs. 15.4 months (HR 0.90, 95% CI 0.59–1.38). At 12 months, cumulative incidence of interventions for CNS disease was 25.5% for the N+C group vs. 36.0% for the L+C group, and cumulative incidence of progressive CNS disease was 26.2% versus 41.6%, respectively. In patients with target CNS lesions at baseline (*n* = 32), confirmed intracranial ORR (iORR) were 26.3% and 15.4%, respectively.

A third HER2 inhibitor, tucatinib, demonstrated excellent antitumor activity in combination with trastuzumab and capecitabine in HER2-positive breast cancer patients with IMD, compared to trastuzumab and capecitabine alone (iORR 47.3% vs. 20%, p=0.03), and prolonged median OS (18.1 vs. 12.0 months, HR 0.58, p=0.005) and median PFS (9.9 vs. 4.2 months, HR 0.32, p<0.0001) (111).

Multiple small studies have shown signal for intracranial activity in patients with metastatic HER2-positive breast cancer with the antibody-drug conjugate trastuzumab emtansine (T-DM1) (112, 113); secondary analysis has further shown T-DM1 to improve OS in patients with trastuzumab-resistant advanced metastatic breast cancer and asymptomatic BrM previously treated with radiotherapy, compared with lapatinib plus capecitabine (114). An exploratory final analysis of the ongoing KAMILLA trial, an international, single-arm, open-label, phase IIIb study evaluating the safety and efficacy of T-DM1 in patients with previously treated, HER2-positive advanced breast cancer, showed a high CNS-specific ORR, including a CNS-specific ORR of ~50% in a subgroup of 67 patients who had not received prior radiation therapy for BrM (115).

Similar to T-DM1, trastuzumab deruxtecan (T-DXd) is an antibody-drug conjugate that has been shown to demonstrate potential therapeutic benefits for HER2-positive breast cancer patients with BrM in early analyses of multiple phase II/III trials, although these analyses have only been reported in abstracts. For example, a preliminary analysis of an ongoing phase II trial, TUXEDO-1, demonstrated an initial iORR of 83.3% in participants enrolled in the first stage of the study (116), while a subgroup analysis of DESTINY-Breast01, another currently active phase II study, found an ORR of 58.3% and median PFS of 18.1 months (95% CI 6.7-18.1) in 24 patients treated with T-DXd (117). Encouraging results were recently presented by Hurvitz and colleagues in a subgroup analysis of DESTINY-Breast03 (NCT03529110), an ongoing phase III trial, comparing T-DXd and T-DM1 in HER2-positive breast cancer patients previously treated with trastuzumab and taxane-based chemotherapy, and we eagerly await publication of their abstract/full-text article.

While the availability of multiple treatment regimens consisting of single and combination agents has broadened the treatment of HER2-positive breast cancer with BrM, most studies in this area have historically excluded IMD patients. In addition to the identification and validation of new agents, current efforts are aimed at understanding the therapeutic efficacy and toxicities of these targeted therapies, and future studies should focus on the possibility of combining these agents with brain-directed radiation therapies to treat BrM.

###### 2.3.1.1.2 Hormone Receptor-Positive Breast Cancer

HR-positive, HER2-negative breast cancers represent a subtype of breast cancers that do not respond to HER2 inhibitors, such as trastuzumab. Historically, treatment of HR-positive breast cancer has been limited to hormonal therapies, including aromatase inhibitors, selective estrogen receptor modulators, and estrogen receptor downregulators. Recent efforts in drug development have identified three cyclin-dependent kinase 4/6 (CDK4/6) inhibitors to enhance the management of HR-positive breast cancer. However, the landmark trials investigating CDK 4/6 inhibitors, such MONARCH 1-3, MONALEESA-2, and PALOMA-1, have excluded patients with BrM (153, 154). Among HR-positive, HER2-negative breast cancer patients with BrM, abemaciclib demonstrated an iORR of 5.2% and median OS of 12.5 months (95% CI 9.3-16.4) in one phase II trial (118). In the same trial, the combination of abemaciclib and trastuzumab did not demonstrate objective intracranial responses in patients with HR-positive, HER2-positive breast cancer. Patients who received abemaciclub and trastuzumab had a median OS of 10.1 months (95% 4.2-14.3) and median iPFS of 2.7 months (95% CI 1.4-4.0) (118). A second CDK4/6 inhibitor, palbociclib, demonstrated an intracranial benefit rate of 53.3% and median OS of 6.4 months (90% CI 2.8-6.8) in a small prospective trial of patients with BrM secondary to breast cancer, melanoma, NSCLC, and esophageal cancer (119). Specific outcomes of the 3/15 patients with HR-positive, HER2-negative breast cancer were not reported, limiting our ability to draw conclusions on the effectiveness of palbociclib in patients with HR-positive, HER2-negative breast cancer. Several clinical trials have been launched to investigate the role of CDK4/6 inhibitors in patients with HR-positive breast cancer patients with BrM (NCT04791384, NCT04923542, and NCT04227327).

###### 2.3.1.1.3 Triple-Negative Breast Cancer

Approximately 50% of patients with TNBC are diagnosed with BrM (155). In this patient population, the survival prognosis following BrM diagnosis remains guarded given the lack of molecular targets compared with HER2-positive and HR-positive breast cancer. Poly(ADP-ribose) polymerase (PARP) inhibitors have been investigated in TNBC patients with BrM. The combination of the PARP inhibitor iniparib and the anti-cancer agent irinotecan demonstrated an iORR of 12.0% and median OS of 7.83 months (95% CI 5.10-10.2) (120). A second PARP inhibitor talazoparib was evaluated in the phase III EMBRACA trial and did not significantly prolong OS in a subgroup of TNBC patients with a history of BrM (HR 0.671, 95% CI 0.366-1.229) (121). Given treatment challenges with targeted therapies, there is a trend toward immunotherapies in TNBC, which will be discussed in the appropriate section of this review.

##### 2.3.1.2 Targeted Therapies in NSCLC

IMD is a frequent complication of NSCLC. Here, we discuss current targeted therapy efforts for three common genetic alterations associated with NSCLC: ALK, EGFR, and Kirsten rat sarcoma virus (KRAS).

###### 2.3.1.2.1 ALK Rearrangements in NSCLC

BrM have been reported to occur in 15-35% of patients with ALK-positive NSCLC, and up to 60% of these patients develop IMD after first-line therapy (156). In the PROFILE 104 trial, the first-generation ALK inhibitor crizotinib improved median PFS in patients with ALK-positive NSCLC with IMD when administered as a single agent compared with platinum-based chemotherapy (9.0 vs. 4.0 months, HR 0.40, p<0.001), however, likely due to low CNS penetration of crizotinib, approximately half of patients suffered CNS progression (122). First line treatment for ALK-positive NSCLC has therefore shifted to next generation ALK TKIs that offer longer PFS and have greater activity within the CNS. In the ASCEND-2 trial, 100/140 enrolled ALK-positive NSCLC patients with IMD and previously treated with crizotinib and another regimen, received ceritinib (123). Of these patients, 33% achieved an ORR with a median PFS of 5.4 months. The median OS for this subset of patients with IMD was not reported (123). Similarly, alectinib was found to demonstrate adequate anti-tumor activity in 52% of crizotinib-resistant, ALK-positive NSCLC patients with IMD in an early single-arm phase II trial (124).

These findings were supported by the ALEX trial, which demonstrated IMD progression in 12% of untreated ALK-positive NSCLC patients with IMD receiving alectinib compared with 45% of patients receiving crozitinb (HR 0.51, p<0.001) (125). In ALK-positive NSCLC patients with measurable IMD and no previous ALK inhibitor treatment, patients randomized to treatment with brigatinib demonstrated an iORR of 78% compared with an iORR of 29% in patients randomized to receive crizotinib (OR 10.42) (126). Collectively, these results suggest that second generation ALK inhibitors effectively reduce IMD progression and death in patients with IMD secondary to ALK-positive NSCLC, compared with crizotinib.

The recent CROWN RCT investigated the third generation ALK inhibitor lorlatinib in ALK-positive NSCLC patients with measurable IMD and no prior systemic therapies. Adequate intracranial anti-tumor activity was reported in 82% of patients receiving lorlatinib compared with 23% of patients receiving crizotinib (OR 16.83) (127). The 12-month iPFS rate among patients treated with lorlatinib was 96%, compared with 60% in patients treated with crizotinib (HR 0.07, 95% CI 0.03-0.l17) (127). Ensartinib, another ALK-inhibitor, has also been demonstrated to have high therapeutic efficacy among patients with ALK-positive NSCLC and BrM in the eXalt3 trial (129). Among patients with baseline BrM, those treated with ensartinib had higher median PFS compared to those treated with crizotinib (11.8 vs. 7.5 months, HR 0.55, 95% 0.30-1.01, p=0.05). A similar trend in median PFS was observed among patients without baseline BrM receiving ersartinib versus crizotinib (NR vs. 16.6 months, HR 0.46, 95% CI 0.27-0.77, p=0.003). Of note, the incidence of BrM was lower in patients receiving ersantinib compared with those receiving crozitinib at 12-months follow-up (cause-specific HR 0.32, 95% 0.16-0.63, p=0.001) (129). These findings suggest that ALK inhibitors may be effective as monotherapies in patients with ALK-positive NSCLC patients and IMD.

Notably, these trials were all designed with intent to allow for study of drug effect on IMD. First, the trials described above allowed entry of patients that had untreated asymptomatic BrM. Second, all trials required that patient undergo MRI of the brain at accrual, regardless of IMD status, then mandated routine surveillance MRI while patients remained on trial. This approach allowed investigators to measure “prevention” of BrM. Finally, all three trials were compared the study drug to crizotinib, a systemically effective agent with poor CNS activity. This design could serve as a model for future trials designed to include the study of intracranial disease.

###### 2.3.1.2.2 EGFR Mutations in NSCLC

Activating mutations in EGFR are present in 14-47% of NSCLC cases, and 2-63% of patients with EGFR-mutant NSCLC develop IMD during the course of their disease, accounting for a significant proportion of NSCLC-IMD cases, especially in East Asian populations (157). The first generation EGFR inhibitors, erlotinib and gefitinib, demonstrated increased efficacy compared with systemic chemotherapy in patients with EGFR-mutant NSCLC (157). Among patients with IMD secondary to EGFR-mutant NSCLC and previous chemotherapy or WBRT, treatment with gefitinib resulted in a partial response in about 10% of patients, and the overall disease control rate and median PFS were found to be 27% and 3 months, respectively (135). Similar results were obtained in subsequent single-arm clinical trials (136–138). Non-comparative trials with EGFR-mutant NSCLC patients with IMD receiving erlotinib or gefitinib as first-line therapy report an iORR of 73.9%, median OS of 18.8 months, and PFS of 7.1 months (139). These results have since been reproduced with no statistically significant differences in OS found between erlotinib and gefitinib (140). The second-generation agent, afatinib, has shown similarly low activity in the CNS.

Of more clinical relevance is the third-generation agent, osimertinib, given its high efficacy in treatment-resistant, EGFR-mutant NSCLC and high CNS activity (158). In EGFR-mutant NSCLC patients with IMD and known or likely resistance to first- and second-generation EGFR inhibitors, osimertinib was found to be more effective than pemetrexed-based chemotherapy regimens (median PFS 8.5 vs. 4.2 months, HR 0.32, 95% CI 0.21-0.49) (141). The benefits of osimertinib were more pronounced in untreated patients with EGFR-mutant NSCLC and IMD when compared with the first-generation EGFR inhibitors erlotinib and gefitinib (median PFS 15.2 vs. 9.6 months, HR 0.47, p<0.001) (142). Given its effectiveness in delaying intracranial progression and death, osimeritinib has become the first-line treatment for patients with EGFR-mutant NSCLC (159).

In addition to osimeritinib, several third-generation agents have been investigated. In two phase I/II trials, lazertinib demonstrated an iORR of 44-85.7% in patients with BrM secondary to EGFR-mutant NSCLC (130, 131). Furmonertinib (formerly, alflutinib) exhibited good treatment efficacy in a phase I/II trial involving 17 EGFR-mutant NSCLC patients with BrM (iORR: 58.8%; median PFS: 9.9 months) (132). These findings were replicated in a phase IIb trial involving 105 EGFR-mutant NSCLC patients with BrM: In 29 patients with one or more measurable BrM, treatment with alflutinib achieved an iORR of 66%, while among the 87 patients in the complete analysis dataset, iORR was 34% and median PFS was 11.6 months (95% CI 8.3-13.8). Across both cohorts, the intracranial disease control rate ranged from 98-100% (133). Several clinical trials have also reported encouraging unpublished results, including a phase 2 expansion of the APOLLO trial (NCT02981108) on the efficacy of almonertinib in metastatic EGFR-mutant NSCLC. Further trials (NCT04808752, NCT04870190) are currently active to clarify the therapeutic efficacy of almonertinib. Third-generation mutant EGFR inhibitors, such as rezivertinib and abivertinib (formerly, avitinib), have been investigated in patients with NSCLC. However, outcomes in subpopulations of patients with BrM have yet to be reported in published formats.

In addition to third-generation EGFR inhibitors, amivantamab, a bispecific antibody directed against EGFR and mesenchymal-epithelial transition (MET) receptor, has been investigated for the treatment of exon 20 insertion-EGFR-mutant NSCLC. Initial phase I trial data from CHRYSALIS was encouraging: an ORR of 39% was achieved in patients with a history of IMD (134). A new clinical trial, MARIPOSA (NCT04487080), has recently been launched to compare the therapeutic efficacy of the combination of amivantamab and lazertinib with the efficacy of osimertinib in patients with untreated EGFR-mutant NSCLC.

##### 2.3.1.3 Emerging Targeted Therapies in NSCLC

Mutations in the proto-oncogene KRAS have been reported in 25-30% of patients with NSCLC and, therefore, represent the most prevalent genomic driver of malignancy in the disease (160). As of December 2021, a single small molecule inhibitor of KRAS, sotorasib, has been developed and approved for the treatment of patients with KRAS^G12C^-positive NSCLC who have previously failed standard therapies; however, its clinical efficacy in the context of IMD remains to be determined (143). Selpercatinib and pralsetinib, inhibitors of the RET pathway, have shown promising systemic and CNS activity in RET fusion-positive disease (144, 145). Repotrectinib and lorlatinib have also been shown to have efficacy and CNS activity in patients with ROS1-positive NSCLC (128, 146). Tepotinib and capmatinib have demonstrated efficacy with good CNS activity in patients in NSCLC with MET exon 14 skipping mutations (161, 162). Finally, larotrectinib exhibited complete or partial intracranial responses in 2/3 patients with BrM secondary to TRK fusion-positive cancers (163). Similar intracranial efficacy was demonstrated with entrectinib in patients with BrM secondary to TRK fusion-positive cancers (iORR 50-62.5%) (164). The therapeutic efficacy of larotrectinib and entrectinib in TRK fusion-positive NSCLC specifically remains unclear, however, since these trials did not stratify patient outcomes by primary cancer type (163, 164).

##### 2.3.1.4 Targeted Therapies in Melanoma

Studies have suggested that half of patients with metastatic melanoma will develop IMD, with a median OS of 4.7 months (165). Of note, genetic alterations in BRAF, including the activating mutations BRAF^V600E^ and BRAF^V600K^, have been identified in 47% of patients with melanoma and in 24% of melanoma patients with IMD, making BRAF mutations a likely target in the treatment of IMD secondary to melanoma (166). In the landmark BREAK-MB trial, dabrafenib, a small molecule inhibitor of BRAF^V600E^, demonstrated adequate anti-tumor activity in 39.2% of BRAF^V600E^-positive melanoma patients with treatment-naïve IMD and 30.8% of BRAF^V600E^-positive melanoma patients with progressive IMD (147). Median OS was 33.1 weeks and 31.4 weeks in BRAF^V600E^-positive melanoma patients with treatment-naïve IMD and progressive IMD, respectively, and median PFS was 16.1 and 16.6 weeks in the same treatment groups. Intracranial response rates, median OS, and median PFS were lower for patients with BRAF^V600K^-positive melanoma with progressive IMD than with treatment-naïve IMD, though no explicit comparisons were made between cohorts (147). A second BRAF^V600^ inhibitor, vemurafenib, demonstrated adequate anti-tumor activity in 18% of patients with BRAF^V600^-positive melanoma with either untreated or progressive IMD (149). Median OS was 8.9 months in patients with untreated IMD and 9.6 months in patients with previously treated IMD, however, no comparisons were made with dabrafenib (149).

In the COMBI-MB trial, the combination of dabrafenib and the mitogen-activated protein kinase kinase (MEK) inhibitor trametinib demonstrated adequate intracranial tumor control in 58% of BRAF^V600E^-positive melanoma patients with treatment-naïve, asymptomatic IMD, 56% of BRAF^V600E^-positive melanoma patients with progressive, asymptomatic IMD, 44% of BRAF^V600D/K/R^-positive melanoma patients with asymptomatic IMD, and 59% of BRAF^V600E/D/K/R^-positive melanoma with symptomatic IMD (148). Median OS was 10.8 and 24.3 months in BRAF^V600E^-positive melanoma patients with treatment-naïve IMD and progressive IMD, respectively. Median PFS was 5.6 and 7.2 months in the same cohorts (148). Several additional single or combination agents, including vemurafenib and the MEK inhibitor cobimetinib or the BRAF inhibitor encorafenib and MEK inhibitor binimetinib, have been described to be effective in treating IMD secondary to BRAF-mutant melanoma. While these discussions have been thus far limited to case series and conference abstracts, early data suggest that multidrug targeted drug regimens for IMD secondary to BRAF-mutant melanoma may be future treatments for patients with BRAF-mutant melanoma and IMD.

#### 2.3.2 Immunotherapies

Immune-checkpoint inhibitors (ICIs) include large monoclonal antibody-based therapies and small molecule inhibitors that upregulate the immune system and its antitumor activity (167, 168). Initial trials with ICIs have supported their use in patients with IMD secondary to NSCLC and melanoma. Additional trials are now underway to study their efficacy in patients with IMD secondary to other primary cancers, including breast cancer. For example, an early phase II trial studying ipilimumab, an anti-cytotoxic T-lymphocyte-associated antigen 4 (CLTA-4) antibody that promotes T lymphocyte destruction of cancer cells, demonstrated that 16% of melanoma patients with asymptomatic IMD receiving ipilimumab and 5% of melanoma patients with symptomatic IMD receiving corticosteroids and ipilimumab achieved an intracranial objective response (169). In the same study, median OS was 7 months in melanoma patients with asymptomatic IMD receiving ipilimumab and 4 months in melanoma patients with symptomatic IMD receiving corticosteroids and ipilimumab.

In addition to CLTA-4, programmed cell death protein (PD-1), which acts as a negative immune inhibitor, has emerged as a potential anti-cancer target. In an early phase II trial studying the anti-PD-1 antibody pembrolizumab, 22% of melanoma patients with IMD and 33% of NSCLC patients with IMD treated with pembrolizumab monotherapy demonstrated an intracranial objective response (170). A slightly lower iORR was found with the administration of the anti-PD-1 antibody nivolumab in patients with NSCLC and IMD (17%) (171). Both studies support the hypothesis that anti-PD-1 antibodies are active in patients with IMD and present future therapy options. The combination of ipilimumab and nivolumab was investigated in Checkmate 204, which demonstrated an iORR in 53.5% of melanoma patients with asymptomatic IMD and 16.7% of melanoma patients with symptomatic IMD. In the same study, median PFS was reported to be 39.3 months and 1.2 months, respectively (172, 173). Finally, the RELATIVITY-047 trial recently reported prolonged PFS in patients with untreated or unresectable melanoma treated with a combination of nivolumab and lymphocyte-activation gene 3 (LAG-3) inhibitor relatlimab compared to those treated with nivolumab alone (HR 0.75, 95% CI 0.62-0.92, p=0.006) (174). A subgroup analysis of patients with BrM and associated intracranial outcomes is necessary to clarify the role of this promising drug combination in the setting of IMD secondary to melanoma.

Monoclonal antibodies against programmed cell death-ligand 1 (PD-L1) have also been investigated. In patients with IMD secondary to TNBC studied in the Impassion130 RCT, the combination of atezolizumab and nab-paclitaxel did not significantly improve median PFS (HR 0.86, 95% CI 0.50-1.49) or median OS (HR 1.34, 95% CI 0.72-2.48) compared with placebo and nab-paclitaxel (175, 176). Additional prospective trials, including NCT03483012 and NCT04303988, may further clarify the role of PD-L1 inhibitors in patients with IMD and TNBC.

Further investigations are necessary to clarify the role of immunotherapy for IMD in patients receiving concurrent targeted therapies (**Table 2**).

**Table 2:** Summary of studies investigating immunotherapies for IMD.

Trial/Study	Drug	Radiation	Study design	Total participants (n)	Cohorts	Median OS (months)	Median PFS (months)	Findings
Margolin et al. (169)	Ipilimumab	–	Single-arm clinical trial	72	Asymptomatic IMD	7	–	iORR: 16% Intracranial DCR: 24%
					Symptomatic IMD + corticosteroids	4	–	iORR: 5% Intracranial DCR: 10%
Goldberg et al. (170)	Pembrolizumab	–	Single-arm clinical trial	52	Melanoma	NR	–	iORR: 22%
					NSCLC	7.7	–	iORR: 33%
Crinò et al. (171)	Nivolumab	–	Single-arm clinical trial	1588	–	8.6	3.0	Overall ORR: 17% Overall DCR: 39%
Flippot et al. (177)	Nivolumab	–	Single-arm clinical trial	73	Untreated IMD	–	2.4	iORR: 12% Intracranial DCR: 50% Intracranial PFS: 2.7 months
					Previously treated IMD (SRS/WBRT)	–	2.5	Intracranial PFS: 4.8 months, HR 0.49, p=0.0277
Tawbi et al. (172)	Ipilimumab + nivolumab	–	Single-arm clinical trial	94	–	–	–	iORR: 55% Intracranial DCR: 57%
Tawbi et al. (173)	Ipilimumab + nivolumab	–	Single-arm clinical trial	165	Asymptomatic IMD	–	39.3	iORR: 53.5% Intracranial DCR: 57.4%
					Symptomatic IMD	–	1.2	iORR: 16.7% Intracranial DCR: 16.7%
Tawbi et al. (174)	Relatlimab + nivolumab	–	RCT	714	Relatlimab + nivolumab	–	–	–
					Nivolumab	–	–	–
Schmid et al. (175)	Atezolizumab + nab-paclitaxel	–	RCT	451	Atezolizumab + nab-paclitaxel	–	4.9 (HR 0.86, 95% CI 0.50-1.49)	–
					Placebo + nab-paclitaxel	–	4.4	–
Schmid et al. (176)	Atezolizumab + nab-paclitaxel	–	RCT	902	Atezolizumab + nab-paclitaxel	14.3 (HR 1.34, 95% CI 0.72-2.48)	–	–
					Placebo + nab-paclitaxel	16.2	–	–
Knisely et al. (178)	Ipilimumab	SRS	Retrospective cohort study	77	SRS + ipilimumab	21.3	–	–
					SRS only	4.9 (HR 0.48, p=0.03)	–	–
Silk et al. (179)	Ipilimumab	SRS	Retrospective cohort study	70	SRS + ipilimumab	18.3	2.7	–
					SRS only	5.3 (HR 0.43, p=0.005)	3.3	–
Mathew et al. (180)	Ipilimumab	SRS	Retrospective cohort study	58	SRS + ipilimumab	56% in 6 months	–	–
					SRS only	45% in 6 months (p=0.18)	–	–
Minniti et al. (181)	Nivolumab or ipilimumab	SRS	Retrospective cohort study	80	Nivolumab + SRS	22.0	10	iORR: 76%
					Ipilimumab + SRS	14.7 (p=0.015)	6 (p=0.02)	iORR: 60%

Median overall survival marked with a dash if the data was 1) not reported or 2) reported for the entire population, including patients without IMD.

CI, confidence interval; DCR, disease control rate; HR, hazard ratio; IMD, intracranial metastatic disease; iORR, intracranial ORR; NR, not yet reached; NSCLC, non-small cell lung cancer; ORR, objective response rate; OS, overall survival; PFS, progression-free survival; RCT, randomized control trial; SRS, stereotactic radiosurgery; WBRT, whole-brain radiotherapy.

#### 2.3.3 Immunotherapy in Combination With Radiation Therapy

Results from studies investigating the combined administration of radiation and immunotherapies suggest a biological synergy between the two modalities (182, 183). Yet, evidence on the treatment of IMD with radiation and ICI is conflicting and limited to retrospective analyses. Knisley et al., for example, reported a median OS of 21.3 months in 27 patients with melanoma and BrM who received ipilimumab in combination with radiosurgery, compared with 4.9 months in 50 patients treated with radiosurgery alone (p=0.03) (178). These findings have been corroborated in another single-institution case series (median OS 18.3 vs. 5.3 months, HR 0.43, p=0.005) (179). On the other hand, investigators from New York University found no improvement in median OS when SRS was administered together with ipilimumab (n=25) compared with SRS as a stand-alone therapy (n=33) in patients with patients with melanoma and BrM (180). Several case reports also describe the combined administration of WBRT and immunotherapies, but evidence from larger cohort studies or RCTs is lacking (184, 185).

There is emerging evidence supporting the use of SRS and nivolumab: Minniti and colleagues reported that nivolumab was more effective in preventing intracranial disease progression and prolonging OS than ipilimumab when either agent was combined with SRS (median PFS 10 vs. 6 months, p=0.02; median OS 22.0 vs. 14.7 months, p=0.015) (181). In a study of patients with renal cell carcinoma and IMD, the PFS benefits of nivolumab were enhanced by prior SRS or WBRT (median iPFS 2.7 vs. 4.8 months, HR 0.49, p=0.0277) (177). Caution needs to be exercised around the current level of evidence, however. One 2013 RCT for example that compared WBRT plus SRS alone versus WBRT plus SRS in combination with temozolomide or erlotinib for NSCLC patients with limited number of metastases showed higher rates of grade 3 to 5 toxicities in the combination arms (11%, 41%, and 49%, respectively, p<0.001) (186). Moreover, BrM may harbour a distinct set of genetic alterations compared with the primary lesion, and thus responses to targeted therapies may be limited (187). Given the success of immunotherapies in the treatment of IMD and the already-established utility of radiation therapy, these combined approaches are promising avenues for the future. Further research is required before these approaches can be reliably translated into clinical practice (**Table 2**).

## 3 Emerging Considerations for Secondary Prevention

Given its impact on survival and QoL, there is clinical interest in early identification of IMD in patients with high-risk primary cancer types. Understanding the pathobiology of IMD may enable the development of new clinical tools to detect early IMD and initiate appropriate treatments in patients with lower systemic metastatic disease burden.

### 3.1 Screening for IMD

Experience from screening efforts for breast, prostate, and lung cancer have shown that early cancer detection and treatment results in improved disease control and prolonged survival (188–190). To date, IMD diagnosis has depended on imaging, either for staging or screening purposes, or to assess patients who manifest neurological symptoms concerning for brain metastases (7). Default intracranial imaging for all cancer patients would be structurally and financially infeasible; further, given the finding in observational studies that nearly 4% of asymptomatic individuals harbor an intracranial “incidentaloma”, this approach would likely lead to overdiagnosis and unintentional overtreatment (191, 192). Instead, many current efforts have focused on screening of patients who are at high risk for the development of IMD (193). A recent review of studies reporting on the incidence of IMD in patients with metastatic and non-metastatic breast cancer found a significantly higher incidence of IMD in patients with metastatic HER2-positive (22-36%) and metastatic TNBC (15-37%) compared with other forms of metastatic breast cancer (approximately 10%) and non-metastatic breast cancer (annual incidence of IMD as first site of recurrence ≤3% for all identified studies) (193). Findings from this review indicate that patients with metastatic HER2-positive and TNBC are at a sufficiently high risk for development of IMD to warrant routine screening. The American Society of Clinical Oncology (ASCO) and the NCCN currently do not recommend routine screening for IMD in women with metastatic breast cancer; conversely, the 2021 European Association of Neuro-Oncology (EANO)/European Society for Medical Oncology (ESMO) guidelines indicate that screening at diagnosis is “potentially justified” in patients with metastatic HER2-positive and TNBC [EANO: IV, n/a; ESMO: IV, B] (194). To clarify the risks and benefits of routine screening in these patients, multiple studies are currently randomizing patients with HER2-positive or TNBC, both subgroups with a well-defined higher risk of IMD, to either receive regularly scheduled MRI brain imaging or standard of care alone (NCT03881605; NCT04030507; NCT03617341) (193, 195). It remains to be determined if these efforts will result in improved outcomes for patients.

Overall, the reliance on imaging, and particularly MRI, has been a profound limitation to efforts for IMD screening, early detection, and treatment. There has been significant interest in development tools for IMD screening that circumvent intracranial imaging, for example, liquid biopsy (196). The concept of liquid biopsy rests on the assumption that different elements of tumor material, such as tumor-specific DNA, RNA, proteins, and exosomes, and circulating tumor cells, can be identified in blood or cerebrospinal fluid (CSF) as a surrogate for cancer burden. To date, these efforts have been limited by the need to isolate and enrich cancer markers in blood to enable sequencing for genomic approaches, which results in technical error and false biological signals, leading to high false-negative results. There are also valid concerns that spillage of intracranial tumor into the systemic circulation may be limited, which could mandate study of CSF rather than blood (a much more invasive process which requires lumbar puncture) to assess intracranial disease (197).

### 3.2 Understanding the Biology of Brain Metastases

There has been significant interest in delineating the biological processes that underlie metastatic progression in cancer and, in particular, mediate the development of IMD (198). Multiple studies have shown that BrM are derived from cancer subclones that are distinct from dominant populations in the systemic cancer cell pool (199, 200). This finding raises the possibility that primary tumor profiling could identify patients who harbor subclones that are organotypic for the brain and are thus at risk of IMD (201). Further, this finding promises the likelihood of novel pathways, both cell-intrinsic and environmental, that are critical for IMD development and that could be targets for IMD prevention (187, 202, 203).

## 4 Conclusion

Diagnosis of IMD places a significant burden on patient survival and QoL. Over the past several decades, technical innovations and an advancing understanding of tumor biology have enabled physicians to optimize treatment outcomes for these patients. While WBRT initially formed the mainstay of localized treatment for IMD, surgical resection and SRS have become established treatment approaches for patients with limited intracranial disease burden and are increasingly considered for a wider patient spectrum. Several molecular drivers have also been identified as targets for systemic therapy. While most of these agents historically had limited application for treatment of IMD, mounting evidence suggests that some targeted therapy drugs may retain activity in the brain, especially in patients with IMD due to single driver-mediated breast cancer, NSCLC, and melanoma. Early investigations into the efficacy of immunotherapies and their combination with radiation therapy may further form future avenues of treatment. These advances promise to improve outcomes patients with cancer and IMD.

## Author Contributions

AL and KG contributed equally to the design and writing of the manuscript and share first authorship. SD supervised this work. All authors contributed to the article and approved the submitted version.

## Funding

KG is supported by the Graduate Diploma in Health Research at the University of Toronto. SD is supported by the Canadian Institute for Health Research and Gratitude 10. The funding source had no role in the preparation and submission of this review article.

## Conflict of Interest

SD acts as a consultant for Medexus and is on the advisory board for the Subcortical Surgery Group and Xpan Medical as well as the Speaker’s Board for the Congress of Neurological Surgeons, American Association of Neurological Surgeons, and Society for NeuroOncology. KJ is a speaker/advisor board/consultant for: Amgen, Astra Zeneca, Apo Biologix, Eli Lilly, Esai, Exact Sciences, Knight Therapeutics, Pfizer, Merck, Novartis, Purdue Pharma, Roche, Seagen, Viatris; and receives research funding from Eli Lilly, Astra Zeneca.

The remaining authors declare that the research was conducted in the absence of any commercial or financial relationships that could be construed as a potential conflict of interest.

## Publisher’s Note

All claims expressed in this article are solely those of the authors and do not necessarily represent those of their affiliated organizations, or those of the publisher, the editors and the reviewers. Any product that may be evaluated in this article, or claim that may be made by its manufacturer, is not guaranteed or endorsed by the publisher.
